# Management strategies for isolated premature thelarche: a risk-stratified clinical pathway favoring “watchful waiting”

**DOI:** 10.3389/fendo.2025.1705194

**Published:** 2026-01-26

**Authors:** Yan Xu, Hongtao Cui, Xinna Wang, Fang Liu

**Affiliations:** 1 Department of Pediatrics, First Affiliated Hospital, Henan University of Chinese Medicine, Zhengzhou, Henan, China; 2 School of Pediatrics, Henan University of Chinese Medicine, Zhengzhou, Henan, China; 3 Department of Pediatrics, Chongqing Traditional Chinese Medicine Hospital, The First Affiliated Hospital of Chongqing University of Chinese Medicine, Chongqing, China; 4 Department of Encephalopathy, Affiliated Hospital of Changchun University of Chinese Medicine, Changchun, China; 5 Department of Endocrinology and Metabolism, Chongqing Traditional Chinese Medicine Hospital, The First Affiliated Hospital of Chongqing University of Chinese Medicine, Chongqing, China

**Keywords:** isolated premature thelarche, watchful waiting, active intervention, risk stratification, central precocious puberty

## Abstract

Isolated Premature Thelarche (PT) is a common clinical issue in pediatric endocrinology. Because its early presentation resembles that of Central Precocious Puberty (CPP), which requires intervention, its management is highly controversial, caught between “active intervention” and “watchful waiting.” This leads to inconsistent clinical practices and significant anxiety for affected families. This review aims to move beyond this traditional dichotomy by conducting an in-depth analysis of the natural history, pathophysiological mechanisms, diagnostic criteria, and progression risks of PT. We propose a new paradigm for a tiered, individualized management approach based on risk stratification, providing a clear, evidence-based pathway for clinical decision-making. Through a comprehensive review of recent clinical studies, reviews, and expert consensus, this paper analyzes the theoretical basis and evidence supporting a “watchful waiting” strategy. It defines the rigorous initial evaluation and dynamic monitoring protocols required for its implementation and clarifies the quantitative indicators for transitioning from “observation” to “active intervention.” For children with typical PT—characterized by a normal growth rate, no significant bone age advancement, a prepubertal pelvic ultrasound, and a very low basal luteinizing hormone level—”watchful waiting” or “active surveillance” is a safe, effective, and preferred management strategy. This is an active medical monitoring process, not passive waiting. “Active intervention” (i.e., Gonadotropin-Releasing Hormone agonist therapy) should be strictly limited to the few “progressive” cases that show sustained pubertal progression, growth acceleration, and significant bone age advancement during follow-up, and are confirmed as CPP by a Gonadotropin-Releasing Hormone stimulation test. The risk-stratified management pathway proposed herein aims to effectively address the clinical dilemma, avoid over-treatment, and achieve individualized, precise management for children with PT.

## 1 Introduction

Isolated Premature Thelarche (PT) is defined as the isolated development of breast tissue in girls before the age of 8, without other signs of secondary sexual characteristics (such as pubic or axillary hair) or growth acceleration. It is one of the most common variants of precocious puberty in childhood (1). Epidemiological data show significant geographical and temporal variations in the incidence of PT. For instance, a study in Southern China reported a prevalence of 4.8% among girls aged 2 to 7 years (2), whereas a 20-year nationwide study in Denmark observed a significant increasing trend in the diagnosis of all forms of early puberty, including PT (3). Although PT is generally considered a benign condition, its clinical presentation is remarkably similar to that of Central Precocious Puberty (CPP), which requires timely intervention, creating a core challenge in clinical diagnosis and decision-making (4).

In clinical practice, clinicians and parents of children with PT often face a dilemma. On one hand, concerns about the potential impact of early puberty on final height, the risk of psychological problems, or the evolution into true precocious puberty often drive families to seek and push for more “active” investigations and even treatment (5). On the other hand, a large body of evidence from longitudinal follow-up studies indicates that the natural history of the vast majority of PT cases is benign. A follow-up study of 158 girls with PT found that breast development completely regressed in 24.7% of cases and remained stable in 32.9% during the follow-up period, confirming its self-limiting or non-progressive nature in over half of the cases (6). Therefore, excessive intervention in all children with PT not only carries risks of unnecessary drug exposure and financial burden but may also cause iatrogenic psychological stress. Currently, there is a lack of globally unified clinical guidelines for the management of PT, leading to wide variations in practice among different medical institutions and clinicians, with both “over-treatment” and “delayed diagnosis” coexisting (7).

The central theme of this paper is to address this clinical dilemma: should PT be managed with “active intervention” or “watchful waiting”? We argue that framing these as mutually exclusive options is inappropriate. This paper aims to advocate for a more scientific and precise perspective: under the premise of accurate diagnosis and dynamic risk stratification, “watchful waiting” should be the mainstream management strategy for PT. At the same time, this paper will delineate clear boundaries and indications for “active intervention.” Clarifying this viewpoint is of significant practical importance for unifying clinical understanding, standardizing diagnostic and therapeutic practices, avoiding over-treatment, and ultimately achieving the best individualized management for every child with PT.

## 2 Methods

This narrative review was conducted through a systematic literature search in PubMed, Embase, and the Cochrane Library for articles published from January 2000 to December 2024. Search terms included: “premature thelarche,” “precocious puberty,” “watchful waiting,” “GnRH agonist,” “bone age,” “pelvic ultrasound,” “luteinizing hormone,” and “risk stratification.” We included clinical trials, cohort studies, systematic reviews, and expert consensus documents. Exclusion criteria were non-English publications, case reports, and studies without clear diagnostic criteria for PT or CPP.

A total of 28 key studies were selected as the references for this narrative synthesis. Data extraction focused on study design, patient characteristics, diagnostic criteria, management strategies, and outcomes. A summary of these key studies is provided in **Supplementary Table 1**.

## 3 Theoretical basis and evidentiary support for the “watchful waiting” strategy

“Watchful waiting,” or more accurately termed “active surveillance,” is not passive inaction but an active medical management strategy based on a profound understanding of the disease’s natural history. The core of this strategy is to accurately identify the few progressive cases that genuinely require intervention through structured follow-up and monitoring, thereby sparing the majority of individuals with benign, non-progressive conditions from unnecessary treatment. The theoretical foundation of this concept is built upon a thorough understanding of the benign natural course of PT and its unique pathophysiological mechanisms (6–8).

### 3.1 The benign natural history of PT is the cornerstone of “watchful waiting”

Numerous longitudinal follow-up studies provide the most robust evidence for the “watchful waiting” strategy (6, 7). These studies consistently reveal that the vast majority of PT cases follow a benign course, with breast development either regressing spontaneously or remaining stable over the long term, without negatively impacting the child’s overall growth and developmental trajectory. For example, a follow-up study of 158 girls with PT showed that during a multi-year observation period, breast development completely regressed in 24.7% of cases and remained stable in another 32.9%, totaling over half of the cohort, which strongly supports its self-limiting or non-progressive nature (6). The rate of spontaneous resolution is even higher in infants and toddlers diagnosed before the age of 3 (9). More importantly, even if breast development persists, the timing of pubertal onset, developmental progression, and final adult height in these children show no significant difference from their peers (7). It is based on this benign natural history that we can confidently assert that universal medical intervention for all children with PT is not cost-effective. This does not, however, mean that the risk of progression can be ignored. Longitudinal follow-up data indicate that a minority of PT cases will eventually progress to CPP requiring treatment (6). Therefore, the core task of clinical management shifts from “whether to treat” to “how to accurately identify this minority of individuals who require treatment.”

### 3.2 Differences in pathophysiology dictate different management strategies

The fundamental difference in pathophysiology between PT and CPP is the primary reason for adopting different management strategies. The core of CPP is the premature and sustained activation of the hypothalamic-pituitary-gonadal axis (HPGA), leading to a progressive, pulsatile secretion of gonadotropins [especially Luteinizing Hormone (LH)]. This drives full gonadal development and a continuous rise in sex hormone levels, ultimately resulting in rapid bone age advancement and growth acceleration (7). In contrast, the mechanisms underlying PT are more diverse, with the common feature being the absence of sustained HPGA activation. Currently accepted mechanisms include (1): Transient HPGA activation or imbalance: Especially during the first two years of life, the HPGA undergoes a period of physiological activation known as “mini-puberty.” Some PT cases may be related to a higher degree of activation or a delayed quiescence of the HPGA during this phase. Gonadotropin secretion during this period often shows a follicle-stimulating hormone (FSH)-dominant response, rather than the typical LH-dominant pulsatile secretion seen in CPP, a pattern insufficient to drive full pubertal progression (10). (2) High peripheral tissue sensitivity to estrogen: The breast tissue of some children may exhibit a hypersensitive response to normal physiological concentrations of circulating estrogens (11). (3) Exposure to exogenous estrogens or endocrine-disrupting chemicals (EDCs): Exposure to products containing EDCs is a significant acquired factor in inducing PT. A case-control study clearly demonstrated a significant association between such exposure and the occurrence of PT, involving substances such as phytoestrogens, bisphenol A (BPA), phthalates, and parabens (12). (4) Other rare factors: Such as transient estrogen secretion from small functional ovarian cysts, or syndromes associated with specific gene mutations (e.g., PURA syndrome (13), aromatase excess syndrome (14)). It is precisely because the HPGA is not persistently activated that PT does not lead to rapid bone age advancement and growth acceleration, which fundamentally supports the rationale and safety of the “watchful waiting” strategy.

## 4 Clinical implementation pathway for the “watchful waiting” strategy

To safely and effectively implement the “watchful waiting” strategy, a standardized clinical pathway that includes a rigorous initial evaluation and regular dynamic monitoring must be established. The core objective of this pathway is to accurately screen for low-risk children suitable for observation at the initial assessment and then to promptly identify “red flags” indicating a possible transition to CPP through dynamic follow-up, thereby achieving precise, stratified management.

### 4.1 Initial diagnosis and risk assessment as the starting point of the pathway

The initial diagnosis is the critical first step in distinguishing PT from early CPP and laying the foundation for subsequent management strategies. A comprehensive initial evaluation should include a detailed medical history, a thorough physical examination, and key ancillary tests (5). The history should focus on the onset and progression rate of breast development, any fluctuations, recent height growth, presence of vaginal discharge or bleeding, and any history of exposure to exogenous estrogens or EDCs. The physical examination requires precise measurement of height and weight, plotted on a growth chart to assess growth velocity, along with careful breast palpation to determine the Tanner stage and confirm the absence of other secondary sexual characteristics. Breast ultrasound can serve as an objective adjunct to clinical examination, helping to distinguish true glandular tissue from adipose tissue and staging breast development, particularly in overweight girls or when palpation is equivocal (15). Based on this, key ancillary tests provide objective evidence for risk stratification:

1. Bone Age (BA): Bone age is one of the most important non-invasive tools for differentiating PT from CPP. In typical PT, the BA should be consistent with or only slightly advanced compared to the chronological age (CA), generally defined as a difference (BA-CA) of less than 1 year (5).
2. Pelvic Ultrasound: Pelvic ultrasound provides direct morphological evidence of the developmental status of the uterus and ovaries. In children with PT, the uterus and ovaries should appear prepubertal. Specific indicators for identifying pre-pubertal girls include a uterine length of less than 4 cm, a mean ovarian volume of less than 2 cm³, and a fundo-cervical ratio of less than or equal to 1 (16). The three most efficient parameters for girls aged >6 to 8 years were cervical thickness, cervical volume, and uterine volume, whereas for girls aged >8 to 10 years, they were endometrial thickness, uterine volume, and cervical thickness. Uterine volume was among the most effective parameters for both predicting CPP and for distinguishing CPP from PT (17).
3. Basal Sex Hormone Levels: Serum gonadotropin and estrogen levels are central to the differential diagnosis. In children with PT, serum estradiol (E2) and FSH levels are typically in the low, prepubertal range. Among these, the basal LH level is of the highest diagnostic value. Multiple studies have shown that a very low basal LH level (usually <0.1-0.2 IU/L) is strong evidence supporting a diagnosis of PT and ruling out CPP (18).

Based on this comprehensive assessment, a girl presenting with isolated breast development, a normal growth rate, no significant BA advancement, a prepubertal pelvic ultrasound, and a very low basal LH level can be diagnosed with typical PT. She can then be classified into the low-risk group, and the “watchful waiting” protocol can be formally initiated.

### 4.2 The dynamic follow-up protocol is the core of the pathway

For children placed in the “watchful waiting” cohort, regular dynamic follow-up is the fundamental guarantee of the strategy’s safety. It is recommended to conduct the first follow-up 3 to 6 months after the initial diagnosis, with subsequent visits every 6 to 12 months depending on the child’s condition (6). The core monitoring content should comprehensively cover clinical manifestations and imaging changes, including precise calculation of growth velocity, re-evaluation of breast Tanner stage, and vigilance for any newly emerging secondary sexual characteristics. Furthermore, periodic re-evaluation of BA and pelvic ultrasound (typically every 6 to 12 months) is crucial for dynamically assessing skeletal maturation and the development of internal and external reproductive organs, serving as a key step in the timely identification of “progressive” cases (6). In parallel, family education and shared decision-making play an indispensable role in this process. Physicians need to patiently and clearly explain the benign nature of PT, its common natural course, and the low risk of progression to parents, effectively alleviating their anxiety with scientific explanations. At the same time, parents must be instructed on how to accurately identify the “red flags” that warrant concern (see next section) and educated on how to avoid potential EDCs in the environment. Through this process, parents are transformed from passive, anxious observers into active partners in the monitoring process. This builds mutual trust between the physician and the family, enabling them to jointly make decisions that are in the best long-term interest of the child’s health, which is the core embodiment of modern medical humanistic care and the shared decision-making model (19).

## 5 Clear indications for the “active intervention” strategy

“Active intervention” should not be considered a routine management option for PT. Instead, it is a necessary medical measure specifically reserved for “progressive” cases identified within the “watchful waiting” cohort that have clearly transitioned to CPP. This stratified management ensures that intervention is targeted and necessary, reflecting the principles of precision medicine in the management of PT.

### 5.1 Evidence-based decision points for shifting from “observation” to “intervention”

The transition from “observation” to “intervention” must be based on a series of objective, quantifiable clinical and laboratory evidence, which collectively constitute the “Red Flags” indicating a change in the nature of the condition. The appearance of any one or more of the following indicators during follow-up should raise a high suspicion of progression from PT to CPP, prompting further confirmatory tests and intervention decisions. These signs include: sustained rapid progression of sexual characteristics, such as an advancement of one or more Tanner stages in breast development within 6 months (20); a significant acceleration in growth velocity, with an annual growth rate exceeding two standard deviations (+2SD) above the mean for age, or a clear upward deviation from the original percentile on the growth chart; significant advancement and acceleration of bone age, indicated by a BA exceeding CA by 2 years or more (BA−CA ≥ 2 years), or a disproportionate rate of bone age progression over time (ΔBA/ΔCA > 1) (21); and the appearance of other secondary sexual characteristics, such as pubic hair, axillary hair, or menarche.

In addition to clinical manifestations, evidence from imaging and endocrinology is equally crucial. Pelvic ultrasound examination can be used as an auxiliary means to distinguish normal girls from those with varying degrees of precocious puberty and may serve as a useful preliminary step in selecting patients who require GnRH testing (22). The “gold standard” for final diagnosis and initiation of intervention is a positive Gonadotropin-Releasing Hormone (GnRH) stimulation test. A significant, pulsatile increase in the peak LH level after stimulation, with an absolute value exceeding 5.0 IU/L, is definitive evidence of central, sustained activation of the HPGA. Additionally, a peak LH/FSH ratio greater than 0.6 after stimulation can serve as an important supplementary criterion (23).

### 5.2 Purpose, means, and prudent considerations of intervention

For confirmed progressive CPP, the only widely accepted standard treatment is the use of long-acting Gonadotropin-Releasing Hormone agonists (GnRHa), such as leuprolide or triptorelin (24). It is essential to clarify to parents that the primary goal of GnRHa therapy is not merely to induce breast regression but has more profound objectives. First, by continuously suppressing the prematurely activated HPGA, it effectively delays the premature fusion of the epiphyses, thereby maximizing the improvement and preservation of the child’s final adult height (FAH), which would otherwise be compromised by rapid BA advancement. A comprehensive review indicated that GnRHa treatment initiated in girls before the age of 6-7 can effectively improve their FAH (25). Second, for very young children, delaying pubertal progression can alleviate the psychological stress and social difficulties that may arise from premature physical development, thus promoting healthy psychosocial adaptation.

**Figure 1:**
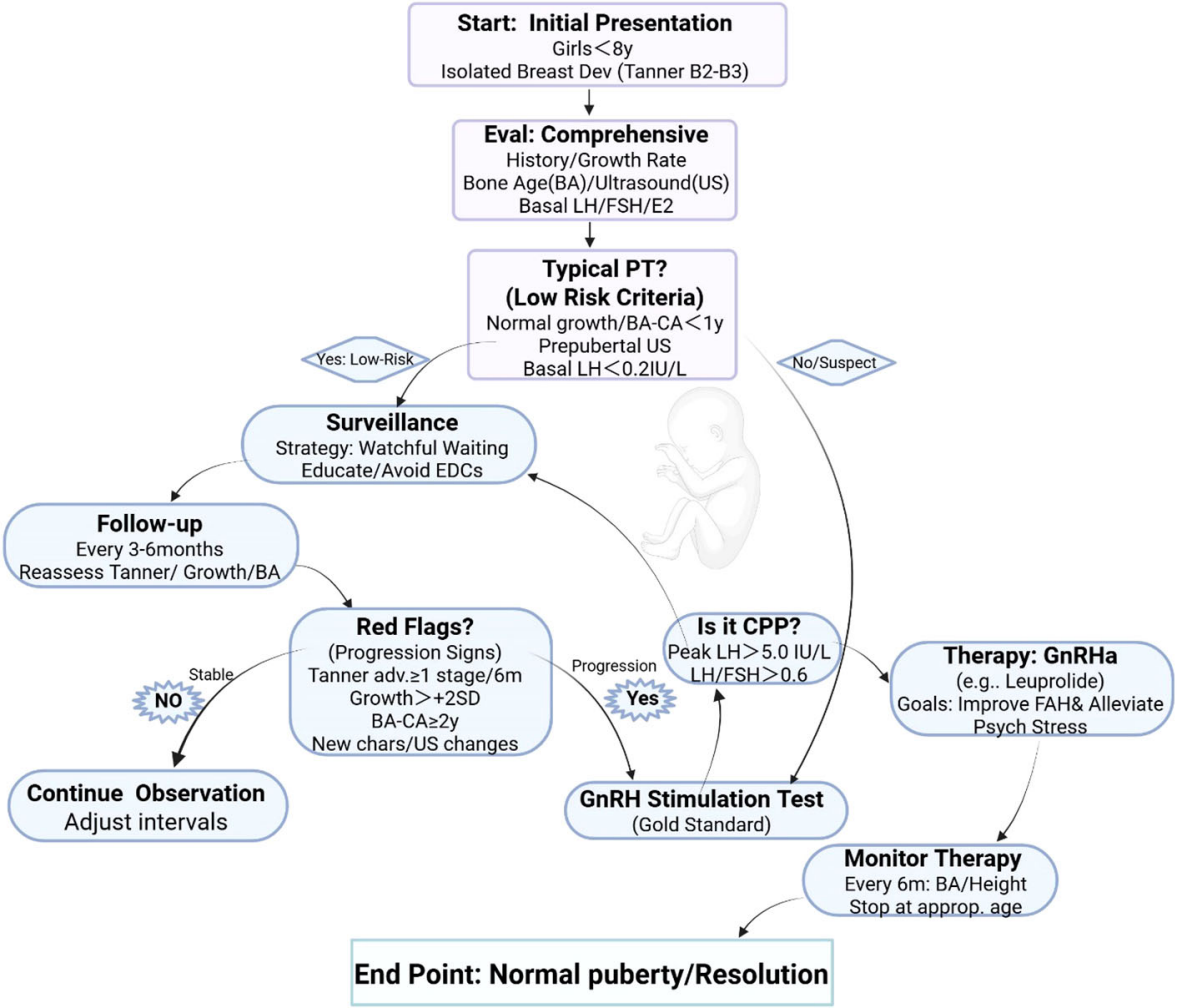
Proposed risk-stratified clinical pathway for the management of isolated premature thelarche. This flowchart outlines an evidence-based, tiered management approach for girls under 8 years with isolated breast development. Initial evaluation distinguishes typical IPT (low-risk: active surveillance) from suspicious cases (GnRH stimulation test). Dynamic follow-up monitors for progression red flags, transitioning to GnRHa therapy only if central precocious puberty (CPP) is confirmed. Shared decision-making and parent education are emphasized throughout. The flowchart employs standardized symbols and colors to represent key elements of the clinical pathway. Rectangles in blue or light blue denote starting/ending points or initial evaluation steps, such as “Start: <8y (Under 8 years) breast dev (Development)” and “Eval: History/BA (Bone Age)/US (Ultrasound)/LH (Luteinizing Hormone)”. Green rectangles indicate surveillance or observation phases, exemplified by “Surveillance: Educate/Avoid EDCs (Endocrine-Disrupting Chemicals)” and “Follow-up: 3-6m (months) reassess”. Red rectangles signify intervention or treatment actions, like “Therapy: GnRHa (Gonadotropin-Releasing Hormone Agonist)/Monitor”. Light purple or gray rectangles represent monitoring or continuation steps, including “Continue: Adjust intervals” and “Monitor: q6m (Every 6 months) BA (Bone Age)/height”. Purple diamonds mark decision points, such as “Typical PT (Premature Thelarche)?”, “Red Flags? Tanner (Staging) adv. (Advance) >=1/6m (months); Growth >+2SD (Standard Deviation); BA (Bone Age)-CA (Chronological Age) >=2y (years); New chars (Characteristics)/US (Ultrasound) changes”, and “CPP (Central Precocious Puberty)? Peak LH (Luteinizing Hormone)>5 IU/L (International Units per Liter); LH (Luteinizing Hormone)/FSH (Follicle-Stimulating Hormone)>0.6”. Solid arrows illustrate the flow direction, including Yes/No branches and cyclic pathways. Abbreviations throughout the figure are interpreted in the following clinical contexts: <8y refers to under 8 years, indicating girls younger than 8 years of age; dev stands for development, specifically breast development; BA means bone age, an assessment of skeletal maturity; US denotes ultrasound, typically pelvic ultrasound examination; LH is luteinizing hormone, a key hormonal indicator; PT abbreviates premature thelarche, relating to isolated premature thelarche (IPT); IPT expands to isolated premature thelarche; CPP is central precocious puberty; GnRH represents gonadotropin-releasing hormone, used in stimulation testing; GnRHa indicates gonadotropin-releasing hormone agonist, a therapeutic agent; EDCs are endocrine-disrupting chemicals, environmental factors to avoid exposure to; IU/L signifies international units per liter, the unit for hormone concentrations; FSH is follicle-stimulating hormone, evaluated in ratio with LH; Tanner refers to Tanner staging for pubertal development phases; adv. means advance, as in Tanner stage advance; SD is standard deviation, for growth velocity exceeding +2SD; CA stands for chronological age, used to calculate BA-CA differences for bone age advancement; chars abbreviates characteristics, denoting new secondary sexual features like pubic hair; FAH is final adult height, a monitoring outcome in therapy; psych denotes psychosocial effects; q6m means every 6 months, specifying follow-up intervals; and approp. is appropriate, referring to the suitable age for discontinuing treatment.

However, initiating GnRHa therapy is a serious medical decision that must be made after a thorough consideration of the benefits and risks. Although GnRHa is generally safe, as a long-term medication, it may be associated with issues such as injection site reactions and short-term effects on bone density and body composition, although existing evidence suggests these effects are mostly reversible after discontinuation of the drug (25). Therefore, strictly adhering to the aforementioned indications and precisely targeting treatment to children with confirmed progressive CPP is a fundamental requirement to avoid over-treatment of the large number of non-progressive PT cases and to protect the health and rights of children, as show in **Figure 1**.

## 6 Discussion and outlook

Through an in-depth review of the management strategies for PT, this paper aims to address the core clinical dilemma raised in the introduction: how to strike a scientific balance between the risk of “over-treatment” and the fear of “delayed diagnosis.” Our conclusion is that abandoning the dichotomous “intervene or wait” mindset and establishing a risk-stratified, tiered management pathway is key to resolving this dilemma. The core of this pathway is to establish “watchful waiting” as the cornerstone strategy for the vast majority of children with typical PT, while setting strict, evidence-based boundaries for “active intervention” in the minority of “progressive” cases.

### 6.1 The risk-stratified pathway is a direct response to the clinical dilemma

The management pathway proposed in this paper is essentially a refined approach to managing clinical uncertainty. By integrating multi-dimensional information from medical history, physical examination, BA, pelvic ultrasound, and basal hormone levels at the initial stage, we can, with a high degree of probability, identify children with typical benign features. This provides a solid initial evidence base for adopting a “watchful waiting” strategy, directly addressing clinicians’ concerns about potential diagnostic delays from a “do-nothing” approach. At the same time, by establishing clear, quantifiable “red flags” during the observation period, we provide a safety net for the timely identification of the few progressive cases. This model of “active surveillance” rather than “passive waiting” allows physicians to confidently explain to anxious parents why medical intervention is not currently necessary, thereby effectively mitigating the tendency for over-treatment driven by uncertainty (5, 8). Therefore, this pathway is not just a technical workflow but also a framework for promoting physician-patient communication, building trust, and facilitating shared decision-making.

### 6.2 The limitation of early accurate prediction is the core challenge

Although the risk-stratified pathway based on dynamic observation is a significant advancement, its fundamental challenge lies in the “time lag” of diagnosis. Currently, we still lack early biological markers that can accurately predict at the onset of PT whether it will progress to CPP. This is particularly difficult in infancy, where the physiological effects of “mini-puberty” can cause a significant overlap in gonadotropin levels between children with PT and early CPP, making early differentiation based on a single time-point hormone measurement especially challenging. Furthermore, increasingly complex environmental and lifestyle factors, such as widespread exposure to EDCs and the rising prevalence of childhood obesity (26), may act as independent or synergistic factors influencing the timing of puberty, further complicating early risk assessment. These factors collectively contribute to the current clinical practice of “wait and see,” which represents a bottleneck that future research urgently needs to address.

### 6.3 Real-world challenges and implementation considerations

While the proposed pathway provides a structured approach, its implementation may face challenges in resource-limited settings, where access to serial bone age assessments or pelvic ultrasound may be constrained. Additionally, managing parental anxiety during the “watchful waiting” period requires effective communication and education, which demands time and counseling skills from clinicians. Future efforts should focus on developing simplified screening tools and telehealth-supported monitoring to enhance feasibility and address these real-world barriers.

### 6.4 Exploring molecular markers and deepening multifactorial research are future breakthrough directions

To overcome the current bottlenecks, future research should focus on two core directions. The first is the exploration of earlier and more sensitive biological markers. The search for novel markers that can provide an early warning of the risk of PT progression at the molecular level is key to achieving truly early and precise prediction. Currently, several hormones that play important roles in HPGA regulation and ovarian function assessment, such as Anti-Müllerian Hormone (AMH), Inhibin B, and Kisspeptin, have shown potential as candidate markers. Preliminary studies have explored their value in differentiating PT from CPP (27, 28). Future large-scale prospective studies are needed to validate their sensitivity and specificity as early predictive indicators, with the aim of advancing risk assessment from macroscopic clinical observation to a more precise molecular subtyping level. The second direction is to conduct large-scale, long-term prospective cohort studies to deepen the understanding of multifactorial interactions. We need to obtain higher-level evidence-based medicine regarding the natural history of PT, the proportions of different subtypes, and the impact of various influencing factors (genetic, environmental, nutritional) on long-term outcomes (such as FAH, reproductive health, and metabolic status). This will not only help to deepen our understanding of the disease mechanisms but may also provide a scientific basis for developing more targeted public health prevention strategies and individualized intervention recommendations.

## 7 Conclusion

In response to the clinical controversy over whether PT should be managed with “active intervention” or “watchful waiting,” the conclusion of this review is clear: abandoning a dichotomous mindset and adopting a risk-stratified, tiered management strategy is key. For the vast majority of children with typical PT confirmed through a rigorous initial evaluation, “watchful waiting” or “active surveillance” is the preferred scientific strategy. This approach is not passive waiting but an active, dynamic monitoring process centered on protecting the child’s long-term health interests. “Active intervention,” namely GnRHa therapy, should be strictly limited to the minority of children confirmed to have progressed to CPP, and its initiation must be based on objective, clear clinical and laboratory evidence. The promotion of this management model will help to standardize clinical practice, effectively manage clinical uncertainty while minimizing over-treatment, and ultimately achieve individualized, precise management for children with PT.

## References

[1] Leung A Lam JM Hon KL . Premature thelarche: an updated review. Curr Pediatr Rev. (2024) 20:500–9. doi: 10.2174/1573396320666230726110658, PMID: 37496240

[2] Zhang J Xu J Liu L Xu X Shu X Yang Z . The prevalence of premature thelarche in girls and gynecomastia in boys and the associated factors in children in Southern China. BMC Pediatr. (2019) 19:107. doi: 10.1186/s12887-019-1426-6, PMID: 30975105 PMC6458611

[3] Brauner EV Busch AS Eckert-Lind C Koch T Hickey M Juul A . Trends in the incidence of central precocious puberty and normal variant puberty among children in Denmark, 1998 to 2017. JAMA Netw Open. (2020) 3:e2015665. doi: 10.1001/jamanetworkopen.2020.15665, PMID: 33044548 PMC7550972

[4] Sultan C Gaspari L Maimoun L Kalfa N Paris F . Disorders of puberty. Best Pract Res Clin Obstet Gynaecol. (2018) 48:62–89. doi: 10.1016/j.bpobgyn.2017.11.004, PMID: 29422239

[5] Kaplowitz P Bloch C . Evaluation and referral of children with signs of early puberty. Pediatrics. (2016) 137(1):e20153732. doi: 10.1542/peds.2015-3732, PMID: 26668298

[6] Cicek D Savas-Erdeve S Cetinkaya S Aycan Z . Clinical follow-up data and the rate of development of precocious and rapidly progressive puberty in patients with premature thelarche. J Pediatr Endocrinol Metab. (2018) 31:305–12. doi: 10.1515/jpem-2017-0247, PMID: 29373318

[7] Brito VN Spinola-Castro AM Kochi C Kopacek C Silva PC Guerra-Junior G . Central precocious puberty: revisiting the diagnosis and therapeutic management. Arch Endocrinol Metab. (2016) 60:163–72. doi: 10.1590/2359-3997000000144, PMID: 27191050

[8] Kaplowitz PB . For premature thelarche and premature adrenarche, the case for waiting before testing. Horm Res Paediatr. (2020) 93:573–6. doi: 10.1159/000512764, PMID: 33352558

[9] Kaplowitz PB Lee PA . Females with breast development before three years of age. Endocrinol Metab Clin North Am. (2024) 53:195–201. doi: 10.1016/j.ecl.2024.01.002, PMID: 38677862

[10] Seymen KG Atar M Cizmecioglu JF Hatun S . Girls with Premature Thelarche Younger than 3 Years of Age May Have Stimulated Luteinizing Hormone Greater than 10 IU/L. J Clin Res Pediatr Endocrinol. (2020) 12:377–82. doi: 10.4274/jcrpe.galenos.2020.2019.0202, PMID: 32349465 PMC7711634

[11] Lee HS Yoon JS So CH Kim KH Hwang JS . No association between estrogen receptor gene polymorphisms and premature thelarche in girls. Gynecol. Endocrinol. (2017) 33:816–8. doi: 10.1080/09513590.2017.1318374, PMID: 28440677

[12] Palmieri FI Gryngarten MG Arcari AJ Umido V Freire AV . Endocrine disruptors as risk factors for idiopathic premature thelarche in girls: A case-control study. Arch Argent Pediatr. (2025) 123:e202410501. doi: 10.5546/aap.2024-10501.eng, PMID: 40168507

[13] Rezkalla J Von Wald T Hansen KA . Premature thelarche and the PURA syndrome. Obstet. Gynecol. (2017) 129:1037–9. doi: 10.1097/AOG.0000000000002047, PMID: 28486374

[14] Giannopoulou EZ Brandt S Zorn S Denzer C von Schnurbein J Fukami M . Long term effects of aromatase inhibitor treatment in patients with aromatase excess syndrome. Front Endocrinol (Lausanne). (2024) 15:1487884. doi: 10.3389/fendo.2024.1487884, PMID: 39634186 PMC11614628

[15] Yuce O Sevinc D . Ultrasonographic assessment of pubertal breast development in obese children: compliance with the clinic. J Pediatr Endocrinol Metab. (2018) 31:137–41. doi: 10.1515/jpem-2017-0243, PMID: 29374763

[16] Dixit M Jaiswal V Usmani Y Chaudhary R . Uterine and ovarian parameters in healthy North Indian girls from 5 to 16 years by ultrasonography. Pediatr Endocrinol Diabetes Metab. (2021) 27:37–41. doi: 10.5114/pedm.2020.103112, PMID: 33599435 PMC10227480

[17] Wen X Wen D Zhang H Zhang H Yang Y . Observational study pelvic ultrasound a useful tool in the diagnosis and differentiation of precocious puberty in Chinese girls. Med (Baltimore). (2018) 97:e0092. doi: 10.1097/MD.0000000000010092, PMID: 29517679 PMC5882436

[18] Chotipakornkul N Onsoi W Numsriskulrat N Aroonparkmongkol S Supornsilchai V Srilanchakon K . The utilization of basal luteinizing hormone in combination with the basal luteinizing hormone and follicle-stimulating hormone ratio as a diagnostic tool for central precocious puberty in girls. Ann Pediatr Endocrinol Metab. (2023) 28:138–43. doi: 10.6065/apem.2346072.036, PMID: 37401058 PMC10329948

[19] Brauner R . Management of premature breast or pubic hair development in young girls. Rev Prat. (2017) 67:386–91., PMID: 30512880

[20] Kilberg MJ Vogiatzi MG . Approach to the patient: central precocious puberty. J Clin Endocrinol Metab. (2023) 108:2115–23. doi: 10.1210/clinem/dgad081, PMID: 36916130

[21] Song X Zhou J Han T Lin Z Chen X Li Y . Early warning value of multiple serum indicators combined with ultrasound detection in girls with central precocious puberty. Front Endocrinol (Lausanne). (2025) 16:1518764. doi: 10.3389/fendo.2025.1518764, PMID: 40260284 PMC12009695

[22] Talarico V Rodio MB Viscomi A Galea E Galati MC Raiola G . The role of pelvic ultrasound for the diagnosis and management of central precocious puberty: An update. Acta BioMed. (2021) 92:e2021480. doi: 10.23750/abm.v92i5.12295, PMID: 34738554 PMC8689311

[23] Vukovic R Milenkovic T Soldatovic I Pekic S Mitrovic K Todorovic S . Triptorelin stimulated luteinizing hormone concentrations for diagnosing central precocious puberty: study of diagnostic accuracy. Endocrine. (2022) 75:934–41. doi: 10.1007/s12020-021-02947-z, PMID: 34826116 PMC8616750

[24] Cheuiche AV Da SL de Paula L Lucena I Silveiro SP . Diagnosis and management of precocious sexual maturation: an updated review. Eur J Pediatr. (2021) 180:3073–87. doi: 10.1007/s00431-021-04022-1, PMID: 33745030

[25] Soliman AT Alaaraj N De Sanctis V Hamed N Alyafei F Ahmed S . Long-term health consequences of central precocious/early puberty (CPP) and treatment with Gn-RH analogue: a short update. Acta BioMed. (2023) 94:e2023222. doi: 10.23750/abm.v94i6.15316, PMID: 38054666 PMC10734238

[26] Leonardi A Cofini M Rigante D Lucchetti L Cipolla C Penta L . The effect of bisphenol A on puberty: A critical review of the medical literature. Int J Environ Res Public Health. (2017) 14:1044. doi: 10.3390/ijerph14091044, PMID: 28891963 PMC5615581

[27] Xue J Song W Si M Sun C Li K Wang W . Serum kisspeptin and AMH levels are good references for precocious puberty progression. Int J Endocrinol. (2020) 2020:3126309. doi: 10.1155/2020/3126309, PMID: 33293954 PMC7700058

[28] Savas-Erdeve S Sagsak E Keskin M Cetinkaya S Aycan Z . AMH levels in girls with various pubertal problems. J Pediatr Endocrinol Metab. (2017) 30:333–5. doi: 10.1515/jpem-2016-0217, PMID: 28245188

