# Supplementary material for: Management strategies for isolated premature thelarche: a risk-stratified clinical pathway favoring “watchful waiting”
Source: Front Endocrinol (Lausanne). 2026 Jan 26;16:1705194. doi: 10.3389/fendo.2025.1705194 (PMC12883376; doi:10.3389/fendo.2025.1705194)
Supplement: Supplementary file 1 [file Table1.doc]

Supplementary Table 1: Summary of Key Studies on Premature Thelarche and Central Precocious Puberty

| First Author (Year) | Study Design | Patient Population | Key Findings/ Relevance to the Review |
| --- | --- | --- | --- |
| Cicek D (2018) [6] | Retrospective Cohort | 158 girls with PT | Over half of PT cases were self-limited or non-progressive (24.7% regressed, 32.9% stable); progression rate to CPP was 29.7%. Provides key natural history evidence for "watchful waiting." |
| Zhang J (2019) [2] | Cross-sectional Study | Girls in Southern China | Reported a PT prevalence of 4.8% among girls aged 2-7 years, providing important epidemiological context. |
| Brauner EV (2020) [3] | Nationwide Registry Study | Danish children (1998-2017) | Observed a significant increasing trend in diagnoses of all forms of early puberty, including PT, highlighting its growing clinical relevance. |
| Kaplowitz P (2016) [5] | Expert Consensus / Guideline | N/A | Provides a framework for evaluation and referral of children with signs of early puberty, emphasizing avoidance of unnecessary workup for PT. |
| Seymen KG (2020) [10] | Diagnostic Accuracy Study | Girls with PT younger than 3 years | Showed that young girls with PT can have stimulated LH >10 IU/L, highlighting the challenge of distinguishing PT from CPP during "mini-puberty." |
| Wen X (2018) [17] | Observational Study | Chinese girls with precocious puberty | Identified efficient pelvic ultrasound parameters (e.g., uterine volume, cervical thickness) for distinguishing CPP from PT, supporting the use of imaging in diagnosis. |
| Chotipakornkul N (2023) [18] | Diagnostic Accuracy Study | Girls suspected of having CPP | Demonstrated the utility of basal LH level and basal LH/FSH ratio for diagnosing CPP, supporting its role in the initial assessment of PT. |
| Vukovic R (2022) [23] | Diagnostic Accuracy Study | Girls undergoing GnRH stimulation test | Validated the high diagnostic accuracy of the triptorelin-stimulated LH peak for CPP, establishing the diagnostic "gold standard." |
| Xue J (2020) [27] | Exploratory Study | Girls with precocious puberty vs. controls | Investigated Kisspeptin and AMH as potential novel biomarkers for differentiating progressive CPP from non-progressive PT, pointing to future research directions. |
